# Objective measurement in routine care of people with Parkinson’s disease improves outcomes

**DOI:** 10.1038/s41531-018-0046-4

**Published:** 2018-04-03

**Authors:** Parisa Farzanehfar, Holly Woodrow, Michelle Braybrook, Sarah McGregor, Andrew Evans, Frank Nicklason, Malcolm Horne

**Affiliations:** 1 Florey Institute of Neuroscience and Mental Health, The University of Melbourne, Parkville, VIC 3010 Australia; 2 Department of Neurology, St Vincent’s Hospital, Fitzroy, VIC 3065 Australia; 3 Department of Neurology, Royal Melbourne Hospital, Parkville, VIC 3050 Australia; 4 Department of Geriatric Medicine, Royal Hobart Hospital, Hobart, TAS 7000 Australia

## Abstract

It is common in medicine to titrate therapy according to target ranges of objectively measured parameters. Objective measurement of motor function is available for Parkinson’s Disease (PD), making it possible to optimise therapy and clinical outcomes. In this study, an accelerometry based measurement and predefined target ranges were used to assess motor function in a Northern Tasmania PD cohort managed by a Movement Disorder clinic. Approximately 40% (*n* = 103) of the total PD population participated in this study and motor scores were within target in 22%. In the 78% above target, changes in oral therapy were recommended in 74%, Advanced Therapy in 12% and treatment was contraindicated in 9%. Following changes in oral therapy, there was a further objective measurement and clinical consultation to establish whether scores had reached target range: if so subjects left the study, otherwise further changes of therapy were recommended (unless contraindications were present). Seventy-seven cases completed the study, with 48% achieving target (including 22% at outset), Advanced Therapy recommended in 19% and contraindications preventing any change in therapy in 17%. In the 43% of cases in whom oral therapy was changed, total UPDRS improved significantly (effect size = 8) as did the PDQ39 in cases reaching target. NMS Quest and MOCA scores also improved significantly. This study shows that many people in a representative cohort of PD would benefit from objective assessment and treatment of their PD features against a target.

## Motor symptoms: benefits of treating to a target

Measuring motor features of patients with Parkinson’s disease (PD) against a target range can guide medication adjustments and improve disease management. An Australian study led by Malcolm Horne at The University of Melbourne used a movement recording system, the Parkinson’s KinetiGraph (PKG), in 103 patients with PD to determine whether their bradykinesia and dyskinesia were within a predefined target range. They showed that adjustments to the oral medication of 14 patients whose PKG scores were outside the target range at the start of the study significantly improved their motor symptoms and brought them within the target range. These findings highlight how objective measurement of motor features can benefit a proportion of patients with PD and contribute to improve the current standard of care based on clinical judgement and patient history.

## Introduction

Measurement is used to guide management in many aspects of clinical care.^1^ It allows the severity of disease to be measured against normal or therapeutic ranges. If measurements are outside of these ranges, then therapy can be appropriately adjusted or added to bring measurements into the target range. Measurements function are best when they represent the action of effective therapies: e.g., diabetic therapies lower blood sugar, asthma therapies improve peak flow. Target ranges are usually derived from normative data and require evidence that outcomes are superior when these scores are within some specified and measurable range (or target). As evidence accumulates, targets often change to reflect therapeutic goals linked to best outcomes. While being “in target” represents good therapeutic control, as pathology progresses, they may become harder to achieve because of contraindications or resistance to therapy.

In a previous pilot study, we examined the effect of using an objective measurement and targets in Parkinson’s Disease (PD).^2^ We used an objective measurement system known as the Parkinson’s KinetiGraph or PKG (Global Kinetics Corporation^TM^, Australia), and target ranges. People with Parkinson’s Disease were included in that pilot if motor function was considered optimal by both neurologist and the PwP. Implicitly therefore, the neurologists did not consider the PwP to have features requiring treatment nor contraindications to adding treatment, if it were required. However, the study found that by using objective measurement, that bradykinesia, dyskinesia and fluctuations, in 86% of these cases were outside the target range. Furthermore, the study neurologist (who specialised in PD) would also have overlooked the need for change in treatment in 29% of cases without the aid of the PKG (and targets). This was chiefly because PwP in these instances, could not provide a history of the presence of bradykinesia, fluctuations or dyskinesia. This was because they did not identify these features as dose related motor symptoms or in some cases were unaware of their presence (even though their spouse may have noticed their presence) and could not provide the history.

The study neurologist then prescribed therapy to bring the objective measures into the target range, using the PKG as a guide, resulting in a significant improvement in clinical scales.^2^ Thus, the finding of this previous pilot study, was that in a selected group of PwP, there were a large proportion who would benefit from change in therapy if these targets were accepted. However, a shortcoming of this pilot study was that it compared the existing assessment of clinicians (and PwP) that motor function was optimal with the assessment provided by objective measurement (against targets). Undoubtedly, a controlled prospective study comparing treatment to objectively measured targets with best clinical care is required to test this pilot result.

Before proceeding to such a formal controlled study, we wished to examine the overall motor function in a population that reflected the full gamut of PD. There are several reasons that a population representing the full range of PD is necessary. As stated above, various contraindications may prevent the use of therapy to bring scores into “target”. A study of a representative population is required to identify the clinical characteristics of these PwP and their relative proportions within the population. It was also relevant to establish the proportion of PwP whose scores of motor function was outside the target range but would require an infusional therapy or deep brain stimulation (Advanced Therapy) to bring their scores toward the control range. More specifically, one aim of this study was to identify the proportion of PwP in a representative cohort who would benefit from objective assessment and treatment of their PD features against a target range. The second aim was to establish the characteristics of these PwP so as to better design a future controlled study but also to most effectively deploy the use of objective measurement in routine care.

The Northern region of the Australian state of Tasmania was chosen for this study. It is a circumscribed population estimated to have ~270 PwP, and approximately 40% were eligible and agreed to participate in the study described here. Of these PwP, 22% had scores within the target range and were not treated further and 13% had contraindications to increasing medications. We then used a similar approach to the previous pilot study and 44% were treated further (14.5% by referring for advanced Therapies) using their pre-existing state as a comparison. There was a significant improvement in motor scores and quality of life in those who were treated with oral therapies. These findings provide further support that PwP gain benefit when PD management is assisted with objective measurement. It also suggests that routine care and a future trial comparing management aided by objective measurement (and targets) with those managed according to the current standard of care using clinical judgement and history alone, should target PwP aged less than 75, with duration of disease between 4-12 years.

## Results

The demographics of the 103 PwP enroled are shown in Table 1.

**Table 1:** Patients’ demographics and clinical characteristics

	All participants^a^	Controlled	Uncontrolled (*n* = 80)	
	(*N* = 103)	(*N* = 23)	Treatable (*N* = 67)	Contraindicated (*N* = 13)
Age	74 (69–78)	74 (68–78)	74 (68–77)	77 (71–80)
Disease duration	5 (3–10)	4 (1–5)	6 (4–11)	8 (4–12)
H&Y	2 (2–3)	2 (1–3)	2 (2–3)	3 (2–4)
UPDRS I	11 (8–16)	11 (6–18)	10 (8–16)	13 (10–26)
UPDRS II	11 (7–17)	8 (4–12)	11 (9–17)	22 (17–30)
UPDRS III	40 (31–51)	31 (23–40)	41 (32–50)	54 (47–62)
UPDRS IV	3 (0–5)	2 (0–4)	3 (0–5)	4 (0–5)
UPDRS total	65 (50–85)	51 (37–67.2)	65.5 (54–83.2)	94.5 (81.5–109)
LED	650 (425–975)	500 (375–715)	700 (450–1075)	700 (600–987)
PDQ39	33 (17–51)	23 (7–39)	31 (18–50)	55 (39–89)
NMS	10 (8–13)	9 (6–12)	11 (8–14)	12 (8–14)
MOCA	22 (19–25)	22 (19–25)	22 (20–26)	18 (13–23)
BKS	26.2 (21.5–31.8)	22 (20.5–24.6)	26.7 (22–30.9)	34.2 (31.8–39.4)
PTO	60.2 (35–82)	35 (31–51.8)	62.4 (42.6–82)	92 (82–99.3)
DKS	1.6 (0.7–3.8)	2.4 (1.2–4.9)	1.6 (0.7–4.2)	0.7 (0.2–1.1)
FDS	8.3 (6.6–10.7)	9 (7–11)	8.5 (6.6–11.7)	7.2 (5.6–8.6)
PTT	2 (0.7–6.9)	1.2 (0.6–6.9)	3 (0.6–7)	1.5 (1–5.2)
PTI	6.6 (3.3–13.2)	4.3 (2.3–8.9)	6 (3.3–11)	17.1 (12.3–26)

^a^69% male

### Control of motor features within the cohort

#### PwP whose motor features were controlled at the start of the study

**Fig. 1:**
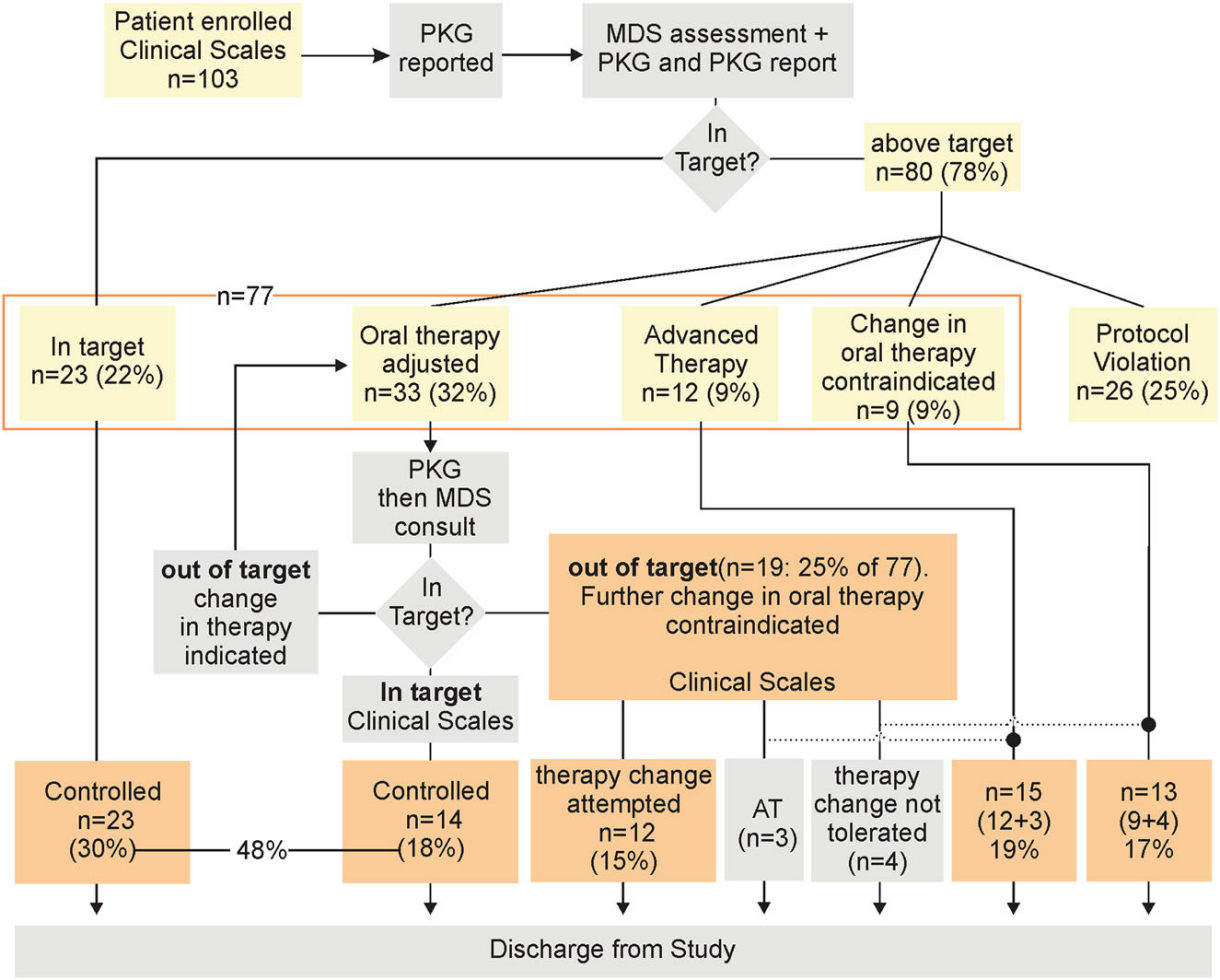
Flow diagram of the study. At the outset of the study, there were 103 participants. The relative proportion of people within target and outside of target are shown in yellow boxes. All proportions in yellow boxes relate to the 103 subjects entering the study. Twenty-two (23%) were controlled and 80 (78%) had uncontrolled motor function, in whom adjustment of oral therapy was attempted in 33 (32%), 12 (12%) were immediately referred for AT, no treatment attempt was made in 9 (9%) cases and 26 (25%) were protocol violations. The 77 PwP participated to the end of the study (i.e., after those classes as protocol violators were removed) have been surrounded by an orange box. The outcomes at the end of the study are shown in orange filled boxes at the bottom of the flow-chart as percentages of the 77 subjects who completed the study. There were 48% who were controlled (c.f. 30% at the outset: 23/77), 15% who were improved but still outside of target, 19% referred for AT and 17% in whom further treatment of motor function was contraindicated

Twenty-three PwP (22%) of the 103 participants had controlled motor function, according to the neurologist’s clinical judgement based on history, examination and inspection of the PKG (Table 2, Fig. 1 and see Methods).

**Table 2:** The state of patients’ symptoms

Symptoms with respect to target range	*N*	PKG findings according to MDS			
		Correct		Incorrect^a^	
		Influence on therapy decision		Influence on therapy decision	
		None	Some	None	Some
Controlled at start of study	23	14	7	2	–
Uncontrolled (78%)					
Controlled by study end	14	1	11	2	–
Not controlled by study end	19^b^	5	13	1	–
Referred for AT by study end	12 (+3^c^)	2	10	–	–
Treatment change contraindicated by study end	9 (+4^c^)	3	5	1	–
Protocol violation	26	5	17	4	–
Total	103	30	63	10	0

^a^3 were due to exercise artifactually raised the dyskinesia scores and 5 cases somnolence caused an increase in the PKG’s bradykinesia scores (BKS) that overestimated the true bradykinesia. In one case the PKG failed to detect truncal dyskinesia and in another case a coarse low frequency tremor resulted in global elevation of the PKG’s dyskinesia score. Note that the PKG reporter identified all of these as artefacts

^b^There were 19 PwP in whom change in oral therapy was attempted but their scores could not be brought into target. Three of these PwP were then at the end of this attempt referred for AT and four cases no change in treatment was possible (see next two rows in Table). All 19 cases were included in assessing improvement from attempting treatment (Table 3)

^c^The numbers outside parentheses are the numbers in these two categories at the start of the study. The numbers in parentheses were the cases reclassified from “Not controlled at the end of study” category by the study end (See Fig. 1)

#### PwP whose motor function were uncontrolled at the start of the study

At the outset of the study 80 (78%) of 103 PwP who participated had uncontrolled motor function (Fig. 1 and Table 2). Adjustment of oral therapy was attempted in 40 of these 80 people, 9 were immediately referred for AT, no attempt was made in 5 cases because of risk of contraindications and 26 did not complete the study (protocol violations). Each of these groups are now described in detail.

#### PwP treated with oral therapy to bring into target range

Thirty-three of the 80 PwP with uncontrolled motor function were treated with oral therapy, and it was possible to bring the motor scores and function under control (i.e., reached to therapeutic targets) in 14 cases (Tables 2 and 3). The median number of visits to achieve control was 3 (2–4): thus 25% required only one adjustment to medication 50% two adjustments and 25% three adjustments. In 19 of the 33 cases, it was not possible to reach therapeutic targets by the end of the study (Tables 2 and 3). The median number of visits to achieve control was again 3, with 65% of case requiring 3 or fewer visits but 15% requiring more than 4 visits. After attempting treatment, 7 of these cases were reclassified: 3 were referred for advanced therapy (AT) and the other 4 were reassigned to the “treatment contraindicated” group because they could not tolerate any change in medications. These 7 subjects were included in the analyses of the effect of attempting a change in therapy (hence *n* = 19 in Table 3). In the remaining 12 cases, some change in therapy was possible although target was not achieved because the study neurologist considered the risks of inducing complications was too great or that the subject was poorly responsive to levodopa or the PwP declining further treatment because of satisfaction with the current state. These patients were not changed to the “treatment contraindicated group” or “protocol violations” because some change in treatment and improvement was possible, compared to the four cases that were reclassified in whom no change was possible.

**Table 3:** Motor and non-motor outcomes in treated subjects

Clinical scores		All treated subjects (*N* = 33)		Optimally controlled (*N* = 14)		Non-optimally controlled (*N* = 19)	
Age		74 (67–80)		73 (65–75)		77 (67–81)	
Disease duration		5 (3–7)		6 (3–8)		4 (3–7)	
H & Y		2 (1–3)		2 (1–2.2)		2 (2–3)	
Number of visits		1		3 (2–3)		3 (2–3)	
Clinical scales		Values	*p* ^a^	Values	*p* ^a^	Values	*p* ^a^
LED	Baseline	600 (438–954)	0.002	600 (394–787)	0.002	625 (450–975)	0.01
	Final	925 (675–1058)		800 (594–1050)		950 (800–1200)	
UPDRS I	Baseline	10 (9–14)	0.0007	10 (9–13)	0.002	10 (8–15)	0.03
	Final	8 (5–12)		8 (5–11)		8 (5–14)	
UPDRS II	Baseline	13 (7–18)	0.03	10 (6–15)	0.1	16 (9–23)	0.1
	Final	9 (4–17)		7 (4–10)		14 (5–21)	
UPDRS III	Baseline	39 (32–49)	0.0009	36 (30–43)	0.04	41 (34–53)	0.009
	Final	36 (28–45)		33 (28–40)		37 (30–48)	
UPDRS IV	Baseline	1 (0–5)	0.01	3 (0–5)	0.007	0 (0–5)	0.5
	Final	1 (0–1.5)		0 (0–1)		1 (0–4)	
Total UPDRS	Baseline	60 (51–86)	0.0001	57 (49–72)	0.002	66 (54–105)	0.0001
	Final	52 (43–77)		48 (39–56)		59 (46–89)	
PDQ39	Baseline	29 (12–45)	0.08	31 (16–44)	0.03	25 (10–49)	0.7
	Final	19 (12–39)		23 (12–37)		17 (11–40)	
NMS ques	Baseline	10 (7–14)	0.02	11 (8–14)	0.03	10 (5–12)	0.3
	Final	10 (4–12)		10 (5–12)		9 (4–14)	
MOCA	Baseline	22 (20–26)	0.02	22 (20–26)	0.3	23 (20–26)	0.05
	Final	24 (22–26)		24 (21–26)		25 (22–26)	
PTI	Baseline	7.2 (5–13.5)	0.1	5.1 (3.4–7.1)	0.1	12.5 (6.4–17.8)	0.6
	Final	6.3 (2.8–12.4)		2.8 (1.6–6.1)		10.2 (5.5–18.6)	
PTT	Baseline	5 (3–13.3)	<0.0001	3.8 (1.3–5.5)	0.01	7 (3–21.9)	0.002
	Final	3 (1.4–6.2)		1.4 (0.7–3.3)		3.9 (1.5–7.8)	
BKS	Baseline	29 (25–33)	0.004	25.6 (23.6–27.9)	0.04	32.5 (28.9–35.9)	0.04
	Final	27 (23.9–31.7)		23.9 (22.4–24.9)		30.3 (27.9–33.8)	
PTO	Baseline	73 (56–88)	0.002	56 (46–71)	0.003	87 (73.5–93)	0.2
	Final	65 (44–85)		44 (37–50)		81 (72–91)	

^a^Wilcoxon matched-pairs test

#### PwP referred for AT

Twelve cases were referred for AT at the first visit and a further 3 during the study after oral therapy was attempted. Thus, by the end of the study, 19% of the 77 PwP who completed the study had been referred for AT. The 12 PwP who were immediately referred were discharged from the study once a decision to refer for AT was made because the study team could not choose or initiate the therapy and the time to follow-up was outside the study timelines. Oral therapy was attempted in 3 cases, but this failed to improve fluctuations and in each of these cases, a pump driven AT was recommended. Although it was a clinical decision to refer for AT, in all cases the PKG showed that the motor function was uncontrolled and in many cases, the PKG report indicated that AT was likely. PKG scores consistent with the need for AT were elevated in each case but this is not described further here as it is the subject of a separate study.^3^

#### Protocol violations

Twenty-six PwP were offered oral therapy but 9 cases declined to accept changes in therapy recommended at the first visit and 17 withdrew during the study, mostly because of intercurrent hospitalisation or travel precluded follow-up examinations. Fifty-eight percent had a H & Y ≥ 3 which may have been a factor in intercurrent illness and unwillingness to participate. All had motor function that would have benefited from treatment changes; e.g., four cases had treatable dyskinesia, one had fluctuations and the remaining 21 cases had treatable bradykinesia. These 26 subjects were removed when assessing the effect of treating to target (*n* = 77).

#### PwP not treated to bring into target range because of contraindications

Initially, there were contraindications (such as orthostatic hypotension, non-responsiveness to L-dopa, cognitive impairment, psychosis and hallucinations) that prevented change in treatment in 9 (Table 2) of the 80 PwP whose symptom control was outside the target range. In a further 4 cases, a change in therapy was recommended but was not tolerated, making 13 cases by the end of the study (17% of 77 who completed the study) where a change in treatment was not attempted or was abandoned because of the emergence of these side effects.

### Clinical characteristics of controlled and uncontrolled PwP

As expected, those PwP whose motor function was not within the target range by PKG criteria and had contraindications to changing treatment had the highest H&Y, UPDRS, BKS and PDQ39 scores (Table 1). They tended to be older, with longer duration of disease, lower MOCA scores and significantly greater daytime somnolence (PTI, measured by the PKG). PwP with motor function already within target at the start of the trial tended to have parameters that were better than the total study population. The PwP whose PKG scores were treatable were intermediate between the other two groups and close to average for the whole study population.

At the commencement of the study, there were 103 participants but there were 26 protocol violations, leaving 77 who completed the study (Fig. 1). At exit, the relative proportions were related to the 77 cases that completed the study (orange boxes, Fig. 1). Thus, at the end of the study there were 48% who were controlled (c.f. 30% at the outset: 23/77), 15% who were improved but still outside of target, 19% referred for AT and 17% in whom further treatment of motor function was contraindicated. Subjects who were controlled had statistically lower UPDRS total, UPDRS III, PDQ39 and NMS scores than either uncontrolled groups (Table 1, Wilcoxon matched-pairs test, statistical data not shown).

### Motor outcomes

Of 33 treated PwP who completed the study and were outside the target, 27 were treated for bradykinesia/wearing off, five for dyskinesia/fluctuations and one for freezing of the gait. Significant improvements were observed in UPDRS II (*p* = 0.03, effect size = 4), UPDRS III (*p* = 0.0009, effect size = 3), UPDRS IV (*p* = 0.01, median did not change) and total UPDRS (*p* < 0.0001, effect size = 8). Moreover, there were significant changes in PKG’s Median Bradykinesia Score (Med BKS: *p* = 0.004, effect size = 2) and PKG’s tremor score (PTT: *p* < 0.0001, effect size = 1.9; Table 3). There was also an improvement in PTO (Percent Time Over target) of 12% between the hours of 09.00–18.00 in those PwP whose scores improved to reach target. This equates to a reduction of 64 min in time outside of target between 09.00–18.00 (or 100 min over the waking time, if this was 14 h).

### Non-motor outcomes

There were statistically significant improvements in UPDRS I (*p* = 0.0007, effect size = 2) and NMS questionnaire (*p* = 0.02). Although improvements were observed in quality of life measured by PDQ39, it was not statistically significant (*p* = 0.08, effect size = 10). However, most PwP (64%) whose PDQ39 did not improve were in fact the same subjects whose PKG scores did not reach target either. Indeed, optimally controlled PwP, who reached target, had a significant improvement in their PDQ39 scores (*p* = 0.03, effect size = 8.5). Interestingly there was a meaningful and statistically significant improvement in cognitive function measured by MOCA (*p* = 0.02, effect size = 2, Table 3).

### Characteristics of non-optimally controlled PwP

Table 3 compares the parameters of PwP who reached target with those who did not. PwP who failed to reach target tended to be older, sleepier in the day time (PTI) and with higher BKS, more tremulous and with more time outside of target range (PTO). While, the median value of many of the clinical scales were remarkably similar there was a much greater spread. For example, although the median PDQ 39 and the MOCA were better in the PwP who failed to reach target, the range included substantial more severe cases (20% with PDQ 39 ≥ 66 and MOCA ≤ 16). Nevertheless, despite failing to reach target, the motor scores improved in most cases (UPDRS III (74% of cases), Med BKS (58% of cases) and PTT (76% of cases)). Interestingly, in some cases PTO improved more than the BKS indicating that PTO, as a measure of the proportion of time that BKS fluctuated outside the target range, can improve more than the median BKS (and UPDRS III) over the day. However, factors measuring quality of life, other than total UPDRS (89% of cases), did not improve as much.

### Changes in the levodopa equivalent dose (LED)

The changes in scores were^4^ achieved through a significant increase in the LED (Table 3). In part this reflects the findings that only 2 of the 33 cases in Table 3 had dyskinesia scores that were outside target. On the other hand, 8 of the 15 subjects referred for AT had dyskinesia outside of the target range for some part of the time between 09.00–18.00. Despite the increase in LED by ~150% in the 33 subjects in Table 3, the UPDRS IV and the median DKS did not increase and in fact was reduced. This indicates that the improvement in bradykinesia was not at the price of increased dyskinesia.

### Concordance between the PKG and the study neurologist

After reviewing the patient in conjunction with the PKG, the study neurologist agreed with the pre-reporting of the PKG in 90% (*N* = 93) of cases (Table 2). In 61% (*N* = 63) of these cases, the PKG added to the clinical findings to an extent that the therapeutic decision was influenced. There was artefactual elevation of the BKS or DKS plot in 10 cases, mostly due to exercise (3 cases) and increased somnolence (5 cases). In 9 cases, these artefacts were noted and reported and thus would not have affected therapeutic decisions. If the PKG had not been acted on without consideration of the interpretation, then it may have led to the changing of a dose at the time that sleep or exercise occurred. A low frequency tremor caused artefactual elevation of the dyskinesia score in one case but this was noted by the PKG reporter. The PKG did fail to detect truncal dyskinesia in one case but this did not alter the therapeutic decision making.

## Discussion

The Northern region of Tasmania has a population of about 150,000 and based on the reported prevalence of PD in Australia,^5^ we estimate the region to have about 270 PwP, of which 233 are enroled with the region’s Movement Disorder Programme with the remaining PwP attending general physicians. The 103 subjects who participated therefore represent ~40% of the PwP in the area, and their clinical scores at entry into the study (Table 1) were consistent with those in other population studies.^6–9^ Furthermore, 88% of the Tasmanian cohort had an H&Y score of III or less, which is only a little more than what is predicted for the overall Australian population (81%).^5^ Most likely the exclusion of subjects in full time residential care accounted for the slightly lower number of H&Y IV&V cases. Thus, the clinical state of the PwP in this cohort at the outset of the study, reflected the clinical outcomes that would be expected from care provided by Movement Disorder Programmes. In support of this, the median PKG scores were almost identical to those of PwP attending other Movement disorder clinics in Australia and somewhat closer to the normal range than those PwP attending specialist Movement Disorder Clinics in the US.^10^ Thus, the proportion of people with “uncontrolled” motor features is likely to reflect that found in most Movement Disorder clinics. We found that 78% (*N* = 80) had “uncontrolled” motor function and a change in treatment was not possible (9%) but was attempted in 69% (*N* = 71). Possibly the proportion with contraindications to change in treatment would have been higher if those PwP in nursing homes had been included in the study.

Importantly however, 52% (when protocol violations are removed and including those referred for AT) did pursue further therapy and in those treated with oral therapy there was a significant improvement in motor, non-motor and quality of life scores. Many of the PwP excluded because of a protocol violation would most likely have benefited from a change of treatment (especially those who resisted a change in therapy or were travelling). However, a key finding of this study is that 78% of PwP in this Movement Disorder Clinic, had scores that were treatable according to the targets set by the PKG. This raises several questions.

### Were the PKG targets meaningful?

The finding that PwP in the controlled range had better motor (UPDRS III) and non-motor (PDQ39, NMS, UPDRS total) scores than those that were uncontrolled argues that there was some validity to these targets. We are not implying that the targets cannot be further refined or optimised through future study- rather that there is reason to argue that the target ranges used in this study reflect less severe PD. Furthermore, shifting the scores of the “uncontrolled” but treatable PwP into the target range resulted in improved motor, non-motor and quality of life and provides further validation that there is meaningful benefit in treating to the target where possible.

### Could improvements have been achieved without targets and objective measurement?

The implications underlying such a question are that this cohort was not being treated to standards of best practice prior to the study and/or; a more experienced clinician could improve clinical care using usual clinical judgement and experience and/or; that very act of scrutiny had a placebo effect that would only be removed by a blinded study. We will consider each of these possibilities. As outlined above, this cohort was managed in a Movement Disorder Programme, the cohort’s scores at entry were similar to those in other Australian Movement Disorder programmes and somewhat less bradykinesia than those in US clinics^10^ and were also similar to those of other published cohort studies.^6–9^ There is thus no reason to believe that their base line standard of care differed from those of other Movement Disorder Programmes. In a previous pilot study of “wearing-off”,^2^ clinicians who specialised in PD failed to recognise “treatable” bradykinesia (according to the definitions used in this study) in 30% of cases and the main reason “wearing-off” or treatable bradykinesia was overlooked was because PwP failed to recognise its presence and report it. We contend therefore that the level of care PwP in this study was consistent with good practice, the PwP were not expressing a need for change and so there would be little reason to expect that a change in therapy would be forthcoming without the impetus provided by objective measurement showing that their scores lay outside a target range. Furthermore, in the current study, the study neurologists acknowledged that objective measurement (in the form of the PKG) contributed to the therapeutic decision in ~80% of cases where treatment was changed (Table 2). Without doubt a randomised trial in which assessments and therapeutic advice with and without access to the PKG would be a superior study. Undoubtedly, there may be a positive effect on both patient and clinician in participating in such a trial. On the other hand, the trial setting does remove some of the opportunities to improve clinical outcomes. For example, we found that some subjects were cautious in accepting the advice of the study neurologist compared to the usual clinician who they had come to know and trust. Some correctable issues, such as addressing orthostatic hypotension before altering PD therapy, were beyond the scope of a clinical trial.

The number of people referred for Advanced Therapy was 14.5% and compares to the 10% in the previous “wearing off” pilot study.^2^ The two study neurologist both work at centres that deploy all three forms of Advanced Therapy and are actively involved in assessing these subjects whereas the clinicians in the Tasmanian Movement Disorder Programme (and in the “wearing off” pilot study^2^) only access these therapies by referral. Thus, this proportion of PwP who are suitable for AT but were otherwise unrecognised, may reflect the difficulty in recognising suitable candidates without the regular experience of working at an Advanced Therapy Centre.

The targets used in this study were motor because the PKG system predominantly measures movement to produce scores for bradykinesia and dyskinesia and also because dopaminergic therapies are thought to mainly target bradykinesia. The greatest improvement was in percent time over the target score (PTO-Table 3), representing about an hour/day (9:00–18:00) with scores outside of the target range. There were similar changes in the UPDRS III and BKS and were smaller than those achieved in a previous pilot study of early fluctuators.^11^ However, there was also improvement in Cognition and Mood, measured by the UPDRS I and MOCA scores, although the change was not significant when measured by NMS Quest. This emphasises that an important component of PD pathophysiology is impaired DA transmission which is manifested by a range of motor but also non motor symptoms and in this respect bradykinesia and dyskinesia may be proxies for the state of dopaminergic transmission. This is reflected in the improvement in Quality of Life scores (PDQ-39), where the effect size was much greater than a minimally significant change in this score^12,13^ and reached or approached statistical significance (depending on the population Table 3).

The fact that altering dopaminergic therapy has an action on both motor and non motor symptoms is relevant to the need for targets. It is difficult at times for both clinician and PwP to recognise which symptoms will respond to changes in dopaminergic transmission. The present findings imply that by improving motor features that there is corresponding change in non-motor symptomatology. The fact that there is also an improvement in PDQ-39 suggest that it is in the PwP’s interests to optimise dopaminergic transmission, even if the motor features are not at the front of the PwP concerns. It is important to underscore that this study relates to the clinical gains that can be made using objective measurement. Bradykinesia is only one manifestation of impaired dopamine transmission but other non-motor features may also appear and can indeed be more troublesome to the PwP.^14^ Mood and affect may change in PwP with fluctuations and dyskinesia, which may in turn influence the objectivity of their reporting.^15^ Clinicians^2,14^ and patients^2^ have difficulty identify fluctuations and it may be that focus on motor dysfunction rather than on all features that respond to dopaminergic stimulation may contribute to this. A recent study^16^ suggests that when placing their current PD symptomatology in the context of the last few months, PwP tend to “normalise” or accommodate to their current state. Considering that PD is fundamentally a disorder of frontal lobe function,^17^ it may be that PwP are less aware than observers of their change in motor and no-motor function. While there may be concerns that OM in PD may impinge on person centred care,^18^ this study suggests to the contrary, that OM provides both the PwP and the clinician with better information to inform decision making. In other diseases, it is well accepted that we should collect objective data around factors that can lead to better outcomes (such as targets for blood sugar) so as inform the individual patient about the benefits and consequences of the various options for treating their blood sugar. In this the patient makes a more informed decision. Indeed, we note that in this study, some PwP elected not to proceed with escalations of therapy even though their scores were outside of target.

Objective measurement is common place in medicine and is almost always used with therapeutic targets. As therapy in PD is currently aimed at modulating dopamine transmission, then objective measurement and targets for routine care should be related to the targets of these therapies and their side effects: in the main, bradykinesia and dyskinesia but also some non-motor features. The objective measure will thus need to quantify the target with sufficient sensitivity to measure the effect of therapy, as well as to capture does related fluctuation in these targets. Even though objective measures such as the UPDRS are available for PD, they have not been deployed in routine care and while diaries capture fluctuations they are cumbersome to use and there are misgivings as to their accuracy.^19^ Both the UPDRS III and the PKG’s score of bradykinesia (BKS) did capture the effect of change in therapy, but the PKG’s measure of fluctuations (PTO) was the most sensitive measure. Thus these measures are sensitive enough to capture changes brought about by therapy that are meaningful enough to improved quality of life rated by patient or carer. The question of whether the targets were appropriate is a separate matter. In other medical conditions, the setting of targets is iterative, with targets changing, usually to become more stringent, if accruing evidence points to optimal outcomes. The targets used here were set as the best estimates of clinicians experienced with PD and with the PKG, but there is every reason that they should be debated and refined with evidence. The improvement in quality of life scores with acquisition of targets suggest that these targets are a reasonable first attempt. The finding that a significant number of people could not have the scores moved into the target range is not in itself an argument that the targets were too stringent: once again other areas of medicine show that with advancing disease it is not always possible to achieve ideal scores without side effects and one would not pursue perfect blood sugars or blood pressures in a patient if the cost was significant side effects. This does not in itself argue against these blood sugar or blood pressure targets.

The aims of this study were to identify the proportion of PwP in a representative cohort who would benefit from objective assessment and treatment of their PD features against a target range and to establish the characteristics of these PwP so as to better design a future controlled study but also to most effectively deploy the use of objective measurement in routine care. The findings do suggest that benefit for PwP can be gained using objective measurement and supports a further study comparing PwP randomised to treatment guided by objective measures with those that are managed according to clinical judgement is now indicated. The findings reported here suggest a future study should focus on PwP aged less than 75 and with duration of disease between 4–12 years.

## Method

This study was conducted in Tasmania, Australia with approval from the St Vincent’s Hospital Melbourne Human Research and Ethics Committee. Written consent was provided by PwP prior to participation.

### Study protocol

#### Study population

Of the ~270 PwP living in the Northern region of Tasmania, 233 are enroled with the region’s Movement Disorder Programme (MDP), run by a geriatrician with experience in PD and two PD nurses. All PwP in the MDP were invited to participate except those living in fulltime resident care (*N* = 12) and; PwP who had already used the PKG as part of their routine care (*N* = 4) and; PwP who had already consulted a Movement Disorder specialist for advanced therapy (*N* = 2) and; 114/153 were willing to be studied further. Nine of the 114 did not have PD and 2 failed to complete enrolment. The remaining 103 PwP were studied according to the following programme:

#### The initial PKG

This was reported without knowledge of the PwP’s condition as controlled (the PKG scores were within the target range) or uncontrolled (some PKG scores were outside the target range). Targets are described below.

#### Initial examination

All 103 PwP were assessed with the following scales: MDS UPDRS, MOCA, NMS and PDQ39. They also received a history and examination by a study neurologist (MH or AE) who recorded a treatment plan before examining the PKG report, as well as making their own assessment of the PKG. The study neurologist then made two key categorisations, taking into account history, clinical findings, contraindications to treatment and the PKG interpretation (Table 2):

#### Were the PwP’s motor features controlled (within the PKG’s target range) or uncontrolled (i.e., outside the target range)?

Note that we use the term “motor features” to refer to bradykinesia and dyskinesia to avoid the terms “signs and/or symptoms”. In making the categorisation of “controlled or uncontrolled”, the neurologist was asked to follow the PKG characterisation of the PwP’s motor features unless there were contraindications to doing so, or if the clinician believed the PKG findings to be incorrect. Thus, the “uncontrolled” group were PwP in whom the PKG scores were outside of the target range and fell into one of the following three cases: i) there was no clinical reason not to do this OR; ii) a change was contraindicated (for example by orthostatic hypotension) OR: iii) the neurologist believed that the PKG findings were at odds with the preferred clinical course.

A change in treatment (when indicated), was achieved by either adjusting oral medication or by referring for advanced therapies (AT). Those PwP referred for AT, or whose motor function was already in target (i.e., “controlled”), or those who had contraindications to adjusting therapy were discharged from the study. The remaining PwP were provided with a plan for change in medications and a follow-up PKG logger (to be worn when the change in therapy would have achieved its intended effect) and prior to the follow up consultation (to assess the effect of the therapy change).

At the follow-up consultation, a decision was made using both history and the PKG findings, as to whether a) optimum control had been achieved; b) whether further changes to therapy were required and were not contraindicated; c) whether further changes to therapy were required but were contraindicated or declined by the patient. If optimal control was achieved, then a final UPDRS, MOCA, NMS and PDQ-39 were arranged. Otherwise the follow up process described above was repeated.

#### What was the value of the PKG in making this decision?

The clinician recorded whether a) they agreed with the pre-study PKG report; b) whether it concorded with what they found on history and examination (i.e., did the PKG add information, misinform or mislead) and c) did the PKG add information that led to clinical decisions that differed to those which would otherwise have been taken. This later was difficult to quantify because information on some occasion altered the therapeutic categorisation (i.e., controlled or not controlled) but in many cases influenced the direction and extent of a decision that may well have been made. These points were described qualitatively (Table 2).

### The PKG system

The PKG system consists of a data logger, which is worn on the wrist of the most affected side, proprietary algorithms that provide bradykinesia and dyskinesia scores every two minutes, and the PKG which is the graphical and numerical presentation of this data. The logger contains a rechargeable battery, a triaxial accelerometer, memory and a capacitive sensor to detect removal from the wrist.^20^ It is worn continuously for 6–7 consecutive days, at the end of which the data is downloaded and analysed using a proprietary algorithm to calculate the following values relevant to this study:

*BKS*: a bradykinesia score (BKS), calculated every two minutes throughout the period of wearing the logger. The median value of these BKS over the period from 09.00–18.00 for the full recording period is known as the median BKS and this correlates with the UPDRS III assessed at the time of doing the PKG.^20,21^

*DKS*: a dyskinesia score (DKS) is calculated every two minutes throughout the period of that the logger is worn. The median value of these DKS over the period from 09.00–18.00 for the full recording period is known as the median DKS and this correlates with the modified Abnormal Involuntary Movement Score assessed at the time of donning the PKG.^20,21^

*PTI*: The Percent Time Immobile over the period from 09.00–18.00. Immobility means that the logger, while being worn by the subject was entirely still for a two-minute period. This has been shown to correlate with the polysomnographic recordings of sleep.^22^

*FDS*: The Fluctuation Dyskinesia Score^23^ estimates the amount of variability in bradykinesia and dyskinesia as measured by the PKG over the course of the 6 days of recording. It provides a measure of the extent of fluctuations in bradykinesia and dyskinesia.

*Percent Time Over Target (PTO)*: This is the amount of time that the BKS was over target and is a representation of “OFF” time in the period from 09.00–18.00 and is the proportion of time that a subject’s BKS is greater than the target used in this study (BKS = 26). The PTO does not include periods of immobility.

*PTT*: The Percent Time Tremor is the proportion of time in the period from 09.00–18.00 that a subject spends with tremor. Tremor is likely to be present if PTT score >1%.^24^

### PKG criteria for the “controlled” and “uncontrolled” states

Target ranges that separate “controlled” PD from “uncontrolled” PD are described below for bradykinesia and dyskinesia. These targets were established by consensus of a panel of four neurologists experienced in treating PD and interpreting the PKG prior to commencing the study.^2^ The targets were based on the bradykinesia score at which most PwP switch from tremor to non-tremor^24^ and the level that approximated 2 h/day of “OFF” time for bradykinesia and 2 h/day with dyskinesia. These PKG criteria target ranges are described below and the reader is referred to the other studies that describe how the PKG scores (BKS, DKS, FDS) are derived and their relationship to other clinical rating scales.^11,20,21^

#### Bradykinesia targets

A BKS = 23 corresponds to a UPDRS III of approximately 20, and BKS = 25 to a UPDRS III of approximately 30. Bradykinesia was considered controlled if the BKS < 23 and uncontrolled if the BKS > 26. Changes in therapy were not required if the BKS < 23. Changes were discretionary for BKS > 23 and < 26. If the median BKS was above 26 or Fluctuation Dyskinesia Score(FDS) < 7.5^6^, then treatment was indicated (unless there was a contraindication to increasing medications). A single dose fluctuation above > 26 was also treated. An FDS > 8.0 was considered controlled.

#### Dyskinesia targets (where DKS represents the median dyskinesia score for the PKG recording period)

Control was defined as a DKS < 9, which corresponds to an Abnormal Involuntary Movement Score (AIMS) of 10. DKS that peaked between 7–9 with an FDS > 13^6^ were treated.

### Statistical method

As many of the populations were not normal distributed, median, interquartile range (IQR) and the Wilcoxon matched-pairs test were used unless otherwise stated.

### Data availability

All data generated or analysed during this study are included in this published article (and its supplementary information files).
